# Coupling and electrical control of structural, orbital and magnetic orders in perovskites

**DOI:** 10.1038/srep15364

**Published:** 2015-10-20

**Authors:** Julien Varignon, Nicholas C. Bristowe, Eric Bousquet, Philippe Ghosez

**Affiliations:** 1 Physique Théorique des Matériaux, Université de Liège (B5), B-4000 Liège, Belgium; 2 Department of Materials, Imperial College London, London SW7 2AZ, UK

## Abstract

Perovskite oxides are already widely used in industry and have huge potential for novel device applications thanks to the rich physical behaviour displayed in these materials. The key to the functional electronic properties exhibited by perovskites is often the so-called Jahn-Teller distortion. For applications, an electrical control of the Jahn-Teller distortions, which is so far out of reach, would therefore be highly desirable. Based on universal symmetry arguments, we determine new lattice mode couplings that can provide exactly this paradigm, and exemplify the effect from first-principles calculations. The proposed mechanism is completely general, however for illustrative purposes, we demonstrate the concept on vanadium based perovskites where we reveal an unprecedented orbital ordering and Jahn-Teller induced ferroelectricity. Thanks to the intimate coupling between Jahn-Teller distortions and electronic degrees of freedom, the electric field control of Jahn-Teller distortions is of general relevance and may find broad interest in various functional devices.

Widespread interest in transition metal perovskite-like oxides over the last several decades can be ascribed to two key discoveries: high-temperature superconductivity in the cuprates and colossal magnetoresistance in the manganites^1^,^2^. Physical behavior exhibited by perovskites is by no means limited to these two phenomena, but also includes ferroelectricity and (anti)ferromagnetism or both simultaneously and coupled in magnetoelectric multiferroics, metal-insulator transitions and thermoelectricity, to name a few. The wide range of functional properties is usually thanks to an interplay between the structural (lattice), electronic (orbital and charge) and magnetic (spin) degrees of freedom allowed within the transition metal oxides^3^,^4^,^5^,^6^. This playground for novel materials physics is not only of fundamental academic interest, but oxide perovskites have already entered industry and have huge potential for novel device applications^7^,^8^.

The possibility of tuning the magnetic properties of a material with an applied electric field has received particular attention for low energy consumption spintronic devices^7^,^9^. In this regard, a promising route to achieve ferroelectricity in magnets is *via* the so-called the hybrid improper ferroelectricity^10^,^11^,^12^. Here, non-polar modes such as antiferrodistortive (AFD) motions, which are ubiquitous in perovskites, eventually combined with antipolar motions, simultaneously drives the polarization and can couple to the magnetic orders^13^,^14^,^15^,^16^,^17^. However, those motions are rather weakly linked to the electronic properties and hence the magnetoelectric coupling is likely not the most efficient. It would be advantageous to replace the AFD or anti-polar motions by another lattice distortion which couples directly to the electronic properties. Such a motion common in perovskites is the Jahn-Teller distortion (JT), and recently, a hybrid improper ferroelectricity mechanism involving such a distortion was reported in metal-organic frameworks^18^,^19^, yielding promising magnetoelectric multiferroic properties.

In an attempt to generalize and extend the concept to any perovskite, we reveal from a universal symmetry analysis that a hybrid improper polarization may arise solely from Jahn-Teller distortions. This mechanism is intrinsic to the perovskite structure and demonstrated here on the vanadate perovskites which exhibit a complex structural ground state including different Jahn-Teller distortions. Using first-principles calculations, we reveal in AA’V_2_O_6_ superlattices an unprecedented orbital ordering and purely Jahn-Teller induced ferroelectricity. We demonstrate that this enables an electric field control of both JT distortions and magnetism. Since JT distortions are intimately connected to electronic degrees of freedom^20^, such as magnetism, orbital orderings and metal-insulator phase transitions to name a few, the proposed mechanism may find broader interest for novel functional devices outside the field of magnetoelectrics.

## Bulk A^3+^V^3+^O_3_

**Figure 1:**
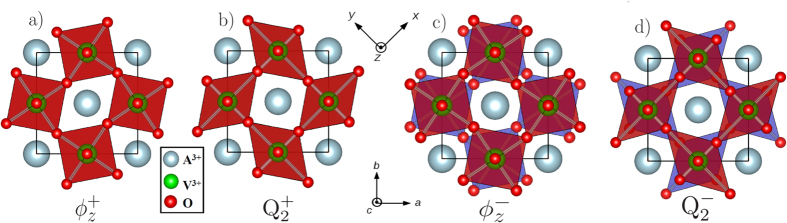
Comparison of JT and AFD motions around the *z* axis; (a) in-phase 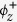 AFD motion (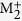 mode); (b) in-phase 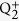 Jahn-Teller motion (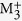 mode); (c) anti-phase 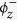 motion (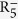 mode); (d) anti-phase 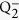 motion (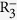 mode). Octahedras for the plane in *z* = 0 are plotted in red and in blue for the plane in *z* = *c*/2. The AFD motions can also appear around the *y* and *z* axes (not shown), whereas the Jahn-Teller motions only manifest around the *z* axis in the vanadates.

Whilst the V^4+^ perovskites (e.g. SrVO_3_^21^) have been studied mainly for their interesting metallic properties, the V^3+^ perovskites are Mott insulators. A^3+^V^3+^O_3_ compounds have attracted much attention since the fifties when they were first synthesized^22^. During this time, many studies began to determine their magnetic, electronic and structural properties^23^,^24^,^25^,^26^,^27^,^28^,^29^,^30^,^31^,^32^,^33^,^34^,^35^,^36^,^37^,^38^,^39^. A central theme at the core of these properties in vanadates is the so-called Jahn-Teller (JT) distortion. The famous Jahn-Teller theorem claims that a material with degenerate electronic states will be unstable towards undergoing a structural distortion lowering its symmetry to remove the electronic degeneracy. In other words, the Jahn-Teller effect is an electronic instability that can cause a structural and metal-insulator phase transition. For instance, in the cubic perovskite symmetry, the crystal field effect splits the *d* electron levels into a lower lying degenerate three-fold *t*_2*g*_ and a higher lying degenerate two-fold *e*_*g*_ state. Hence in 3*d*^2^ systems such as the rare-earth vanadates, a Jahn-Teller distortion is required to split the *t*_2*g*_ levels in order to form a Mott insulating state. We note here the distinction between the Jahn-Teller effect and what we call the Jahn-Teller distortion in this study. Here we define the Jahn-Teller distortion by the symmetry of the atomic distortion as shown in Fig. 1b,d. Whilst a distortion of this symmetry will by definition remove the *d* electronic degeneracy, the origin of such a distortion does not necessarily need to appear from the Jahn-Teller effect. An important result of this study is that the Jahn-Teller distortion can instead be induced by structural anharmonic couplings, being therefore not only restricted to Jahn-Teller active systems^40^.

**Figure 2:**
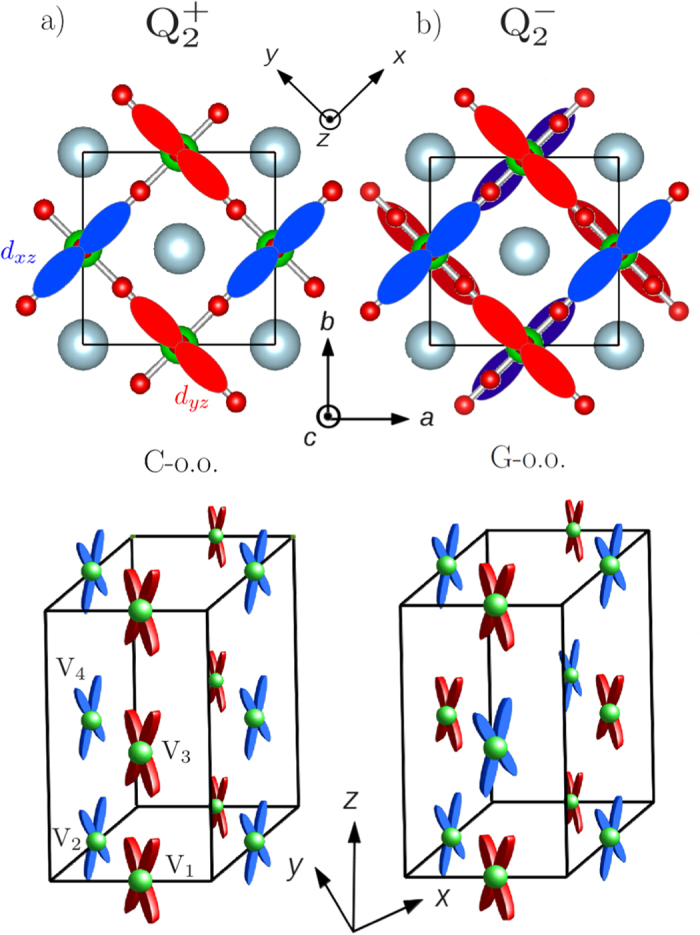
Schematic picture of the idealized orbital orderings between the *d*_*xz*_ (blue) and *d*_*yz*_ (red) orbitals. The *d*_*xy*_ orbital is not shown for clarity. (**a**) Top view of the orbital-ordering induced by a 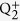 distortion (top). Consecutive planes exhibit identical orbital orderings labelled as a C-type orbital-ordering (C-o.o.) (middle). This results in a G-AFM ordering. (**b**) Orbital-ordering induced by a 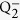 distortion (top). Consecutive planes exhibit out-of phase orbital orderings labelled as a G-type orbital-ordering (G-o.o.) (middle). This results in a C-AFM ordering.

In the vanadates, two different JT distortions are observed^24^,^25^,^26^,^33^, with each one consisting to two V-O bond length contractions and two elongations, often labelled as a Q_2_ distortion^41^. The corresponding distortions are displayed in Fig. 1 where they are compared to the antiferrodistortive (AFD) motions. The AFD motions can be viewed as oxygen octahedra rotations around an axis going through the B cations, while the Jahn-Teller distortions in the present case correspond to oxygen rotations around an axis going through the A cations. Both JT and AFD motions can be either in-phase (Fig. 1.a+b) or anti-phase (Fig. 1.c+d) between consecutive layers and therefore appear at the M or R points of the Brillouin zone respectively. Consequently, we label the Jahn-Teller distortions as 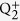 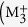 mode) and 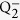 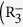 mode). While AFD motions do not distort the BO_6_ octahedra, JT motions lift the degeneracy of the *d* levels through octahedra deformations. According to such distortions, the V^3+^ 3*d*^2^ occupation consists of either a *d*_*xy*_ and *d*_*xz*_ 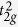 or a *d*_*xy*_ and *d*_*yz*_ 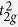 state in an ideal picture. Nearest-neighbor vanadium sites within the (*xy*)-plane develop opposite distortions and hence alternative *d*_*xy*_ and *d*_*xz*_ / *d*_*xy*_ and *d*_*yz*_ occupations as shown in the top panel of Fig. 2. Along the 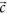 axis, the octahedra deformations and hence orbital ordering are either in phase (C-type orbital order) or anti-phase (G-type orbital order) for the 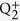 or 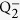 Jahn-Teller distortion respectively (see Fig. 2 bottom panel). Crucially, the orbital ordering determines the magnetic ordering through superexchange interactions^42^,^43^,^44^. Strongly overlapping and parallel orbitals between neighboring sites favors antiferromagnetic superexchange interactions. With this in mind, the 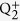 motion favors a purely antiferromagnetic solution called G-AFM whilst the 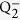 motion favors (*xy*)-plane antiferromagnetic alignment and ferromagnetic out-of-plane alignment called C-AFM. In other words, a C-type orbital ordering (C-o.o.) is linked to a G-type antiferomagnetic ordering (G-AFM), while a G-type orbital ordering (G-o.o.) is linked to a C-type antiferromagnetic ordering (C-AFM). Experiments indeed observe both G-AFM and C-AFM magnetic phases in the vanadates, with each magnetic ordering favoring a certain structural symmetry^25^,^26^,^33^.

At room temperature, all rare-earth A^3+^V^3+^O_3_ vanadates crystallize in a *Pbnm* structure^24^,^25^,^26^,^33^. With decreasing temperature, they undergo an orbital ordering phase transition to a G-type orbital ordered (G-o.o.) phase between 200 K and 150 K (depending on the A-cation size). This transition is accompanied by a symmetry lowering from *Pbnm* to *P*2_1_/*b*. A magnetic phase transition from a paramagnetic to an C-AFM antiferromagnetic state occurs within this phase at a slightly lower temperature between 150 K and 100 K. Finally, for the smallest A cations (A = Yb-Dy, Y), another orbital ordering phase transition to a purely C-type (C-o.o.) arises and is accompanied by a structural phase transition from *P*2_1_/*b* back to *Pbnm*, and a magnetic phase transition from C-AFM to G-AFM. For medium A cations (A = Tb-Nd), a coexistence of *P*2_1_/*b* (G-oo) and *Pbnm* (C-oo) phases is reported^26^,^45^. No further transitions are found for larger A cations (A = Pr, Ce and La).

To better understand the distorted structures of vanadates, we perform a symmetry mode analysis^46^,^47^ of the allowed distortions with respect to a hypothetical cubic phase on three different compounds, covering a wide range of A-cation sizes: YVO_3_, PrVO_3_ and LaVO_3_. The analysis is performed on experimental structural data, and the amplitudes of distortions are summarized in Table 1.

In the *Pbnm* phase (a^−^ a^−^ c^+^ in Glazer’s notations^48^), all three vanadates develop two unique antiferrodistortive (AFD) motions 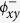 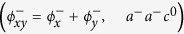 and 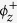 (*a*^0^ *a*^0^ *c*^+^). Table 1 shows that the magnitudes of these AFD motions strengthen with decreasing A-cation size as expected *via* simple steric arguments^49^. Within this *Pbnm* tilt pattern, the 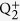 lattice motion is already compatible and does not require any symmetry lowering to appear^38^. This latter observation is in agreement with the sizeable 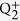 lattice distortion extracted from our analysis on room temperature structures, despite the fact that no orbital-ordering has yet been reported for this temperature range^25^,^26^. The *Pbnm* phase then appears to always be a pure 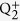 phase. Additionally, an anti-polar 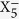 mode whose motion is in the (*xy*)-plane is allowed in the *Pbnm* symmetry (see supplementary materials).

Going to the *P*2_1_/*b* symmetry, a subgroup of *Pbnm*, the aforementioned AFD motions are still present, but the 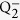 distortion is now allowed and would lead to a G-o.o. phase. However, the *P*2_1_/*b* phase is never an exclusive 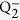 phase but always coexists with the 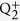 distortion, even for the larger A cations (A = Pr, La). A mixed C-o.o. and G-o.o. should then manifest for all *P*2_1_/*b* structures, independent of orthorhombic/monoclinic phase coexistence. Additionally, another anti-polar 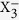 mode, whose motion is now along the *z* direction, arises in this new phase (see supplementary materials).

In order to understand the origin and coupling between these distortions, we can perform a free energy 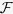 expansion (see methods) around a hypothetical cubic 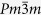 phase with respect to the different distortions. Among all the possible terms in the *Pbnm* phase, two trilinear couplings are identified:
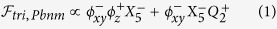
Within the *Pbnm* symmetry, when 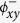 and 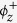 are non zero in magnitude, the free energy of the system is automatically lowered by the appearance of 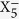 due to the first trilinear term of Eq. 1. Similarly, through the appearence of 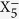, the free energy is again lowered by forcing the appearance of the 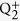 motion thanks to the second trilinear coupling. This explains the presence of the 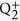 distortion in the *Pbnm* phase of vanadates, even at room temperature. This demonstrates that, in addition to its possible appearance as an electronic instability, it may also appear as a structural anharmonic improper mode within the *Pbnm* phase (whose strength depends on the coupling constant) even in non Jahn-Teller active materials^40^,^50^. Going to the *P*2_1_/*b* phase, two additional trilinear couplings are identified:
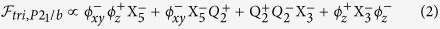
The orbital-ordering phase transition to a G-o.o. phase experimentally observed between 150 K and 200 K for all vanadates^25^,^26^ manifests itself through the appearance of a 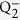 distortion. Consequently, through the third trilinear coupling of Eq. (2), both JT distortions produce the additional anti polar 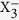 motion. This is in agreement with the experimental data of Table 1. Finally, an extra 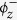 (*a*^0^ *a*^0^ *c*^−^) AFD motion arises in the *P*2_1_/*b* phase through the last trilinear coupling, yielding a rare a^−^ a^−^ c^±^ tilt pattern with both in-phase and out-of phase AFD motion around the 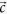 axis. This tilt pattern has previously been predicted to appear within this space group^38^.

Therefore, within this *P*2_1_/*b* phase, both Jahn-Teller distortions coexist, but likely with different origins. The 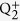 mode is “pinned” into the system through an improper anharmonic coupling with the robust AFD motions while the 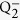 mode may appear through the traditional Jahn-Teller electronic instability. It is interesting to note that this coexistence is allowed due to the improper appearance of 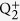, despite there likely being a competition between both JTs. This competition would be understood as an electronic origin to favor one type of orbital ordering over the other, producing a biquadratic coupling with a positive coefficient in the free energy expansion. In the light of there being an abundance of 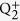 with respect to 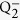 phases across the perovskites, we then propose whether it is this improper appearance of 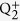 *via* the robust AFD motions that helps favor this phase universally. The vanadates would then be a special case where the 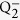 instability is robust enough to appear despite this competition. This universal symmetry analysis and free energy expansion rationalizes the origin of the coexisting orbital ordered phase in the *P*2_1_/*b* symmetry as observed in vanadates both experimentally and theoretically^25^,^26^,^37^,^38^.

The coexistence of both Jahn-Teller motions in the vanadates, will also clearly affect the orbital ordering and consequently the magnetic ordering. One might expect a complex canted magnetic ordering to occur, resembling partly C-AFM and partly G-AFM, as indicated experimentally from neutron scattering on several vanadates. While a pure G-type AFM ordering is observed in the *Pbnm* phase of YVO_3_ with magnetic moments lying along the 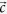 axis, a non-collinear spin arrangement is observed in the *P*2_1_/*b* phase^29^,^30^,^51^,^53^. Indeed, the spin arrangement corresponds to a C-AFM ordering with magnetic moments located in the (*ab*)-plane plus a weaker G-AFM ordering with magnetic moments along the 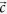 axis. This observation is totally in line with the coexistence of both Jahn-Teller distortions in the *P*2_1_/*b* phase. Indeed, while the *Pbnm* phase can only stabilize a C-o.o./G-AFM ordering, the *P*2_1_/*b* phase has a dominant G-o.o./C-AFM character coexisting with a smaller C-o.o. and consequently a weak G-AFM ordering may appear with magnetic moments along the 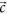 axis^53^. Interestingly, even at the collinear level, our first-principles calculations on the three aforementioned bulk vanadates already indicate this complex magnetic ordering in the *P*2_1_/*b* phase. Within the YVO_3_ *Pbnm* G-AFM C-o.o. ground state, all magnetic sites hold roughly the same magnetic moment (1.811 ± 0.002 *μ*_*B*_) indicating a purely C-o.o./G-AFM ordering as observed experimentally. Going to the *P*2_1_/*b* G-o.o./C-AFM phase of both PrVO_3_ and LaVO_3_, two magnetic sublattices are observed. Indeed, two different magnitudes for the magnetic moments are found in consecutive (*xy*)-VO_2_ layers (1.833 ± 0.001 and 1.826 ± 0.001 for PrVO_3_ and 1.822 ± 0.002 *μ*_*B*_ and 1.813 ± 0.001 *μ*_*B*_ for LaVO_3_) which can be seen as a dominant G-o.o. plus a smaller C-o.o. on the top of the latter one, and consequently a dominant C-AFM plus a weaker G-AFM.

### (AVO_3_)_1_/(A’VO_3_)_1_ layered structures

Magneto-electric multiferroics are widely studied due to their intriguing coupling between ferroelectricity and magnetism (electric field control of magnetism and conversely), and are proposed as promising candidates for lower energy consumption spintronic devices^7^,^9^. However, materials combining both ferroelectric and (anti)-ferromagnetic order parameters are elusive in nature and the identification of new single phase multiferroics remains a challenge for modern day research^54^.

Hybrid improper ferroelectricity, in which a polar distortion is driven by two non-polar motions, emerged recently as a possible new mechanism to induce ferroelectricity in otherwise non-ferroelectric compounds^10^,^11^,^12^,^13^. When considering magnetic compounds, the trilinear coupling between polar and non-polar lattice distortions achieved in such systems appeared moreover as a promising pathway to achieve enhanced magneto-electric coupling^13^,^14^,^18^,^19^,^55^. Rondinelli and Fennie clarified^12^ the emergence of rotationally driven ferroelectricity in ABO_3_/A’BO_3_ superlattices, providing concrete rules for the design of new hybrid improper ferroelectrics.

Following the same spirit, we consider (AVO_3_)_1_/(A’VO_3_)_1_ structures with planes of different A cations layered along the [001] direction. This structure can either appear naturally as in the double perovskites, or through single layer precision epitaxial deposition techniques. The free energy expansion around a *P*_4_/*mmm* layered reference structure (equivalent to 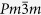 in bulk) then becomes:
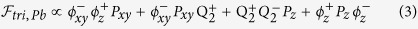


The first observation is that the symmetry breaking due to the A cation layering turns the X antipolar modes to polar modes, i.e. in-plane (110) *P*_*xy*_ and out-of-plane (001) *P*_*z*_^10^,^12^,^54^,^56^,^57^. The first and fourth trilinear couplings of Eq. 3 correspond to the rotationally driven hybrid improper ferroelectricity mechanism^10^,^11^,^12^. The second trilinear term links the in-plane polarization to both an antiferrodistortive (AFD) and Jahn-Teller (JT) distortion, already observed in reference 18. However, we identify in Eq. 3 a new trilinear term 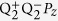 coupling the out-of-plane polarization *P*_*z*_ to both JT distortions. Since JT distortions are intimately connected to orbital-orderings and particular magnetic states as discussed in the previous section, we can expect to have a direct and strong coupling between polarization and magnetism from this term.

In the present work, we have performed first-principles calculations in order to show that (AVO_3_)_1_/(A’VO_3_)_1_ layered structures are indeed ferroelectric and develop both in plane and out-of-plane polarizations. On the one hand, *P*_*xy*_ appears as a slave to the rotations and is indirectly linked to magnetism through the modification of the superexchange path as in the usual rotationally driven ferroelectrics^13^. On the other hand, *P*_*z*_ appears thanks to an electronic instability manifested as a particular orbital and magnetic ordering. Finally, we demonstrate that an electric control of the magnetic state is indeed possible, providing a novel paradigm for the elusive magnetoelectric multiferroics.

In order to test the above hypothesis, we considered two different superlattices: (PrVO_3_)_1_/(LaVO_3_)_1_ (PLVO) and (YVO_3_)_1_/(LaVO_3_)_1_ (YLVO). First principles geometry relaxations (see method section) of the superlattices converged to two metastable states: a C-AFM ordering is found in a *Pb* structure (equivalent to the *P*2_1_/*b* in bulk) while a G-AFM ordering is found in a *Pb*2_1_*m* symmetry (equivalent to *Pbnm* in bulk). We find that PLVO adopts a *Pb* C-AFM ground state while YLVO adopts a *Pb*2_1_*m* G-AFM ground state. The symmetry adapted modes and computed polarizations of all metastable phases are presented in the supplementary material. As predicted, the *Pb*2_1_*m* ground state of YLVO only exhibits a *P*_*xy*_ polarization, whose magnitude is 7.89 *μC*.*cm*^−2^. However, the *Pb* ground state of PLVO develops both *P*_*xy*_ and *P*_*z*_ polarizations of 2.94 and 0.34 *μC*.*cm*^−2^ respectively. The *P*_*z*_ contribution indicates a Jahn-Teller induced ferroelectricity (third term of Eq. 3). Below we explore the origin of *P*_*xy*_ and *P*_*z*_ in more detail.

Bulk vanadates exhibit a *Pbnm* phase at room temperature and hence both superlattices should first go to the equivalent *Pb*2_1_*m* intermediate phase. We therefore begin by providing insight on the driving force yielding the various distortions within this phase. For this purpose, we condense different amplitudes of distortions (see methods) within the metastable *Pb*2_1_*m* state of the PLVO superlattice starting from an ideal *P*_4_/*mmm* structure (for each potential, see supporting information). Four main distortions are then present in this *Pb*2_1_*m* phase: 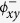, 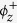, 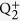 and *P*_*xy*_. As expected, the two antiferrodistortive motions are strongly unstable (approximately 1 eV of energy gains for each) and are the primary order parameters of this *Pb*2_1_*m* symmetry. *P*_*xy*_ and 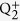 present single wells which are the signature of an improper anharmonic appearance^55^. Therefore, the *P*_*xy*_ polarization appears through a hybrid improper mechanism driven by the two rotations through the first term of Eq. 3. Furthermore, as predicted in the first section, this analysis suggests that the 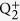 appears with a structural hybrid improper mechanism rather than an electronic instability in this compound.

Having considered the intermediate *Pb*2_1_*m* phase, we next turn our attention to the phase transition of PLVO to its *Pb* ground state. Curiously, a phonon calculation on the intermediate *Pb*2_1_*m* phase did not identify any unstable modes, indicating that no lattice motions can be responsible for the phase transition. Clearly, the system has to switch from G-AFM to C-AFM and therefore in an attempt to understand this phase transition we performed the following two sets of calculations. The atomic positions were fixed to the intermediate *Pb*2_1_*m* structure and the energy was computed i) with imposed and ii) with no imposed, *Pb*2_1_*m* symmetry for the electronic wavefunction, both within the two possible magnetic states. While for the G-AFM calculations, no energy difference is observed between calculations with and without symmetry, the C-AFM calculation with no symmetry leads to a lower energy (around 4.5 meV) than the one with imposed symmetry. The only difference between the two calculations is that the electronic structure is allowed to distort and consequently breaks the symmetry. We discover that, even with the atoms fixed in centrosymmetric positions along the *z* axis, the electronic instability creates an out-of-plane polarization *P*_*z*_ of 0.04 *μC*.*cm*^−2^.

**Figure 3:**
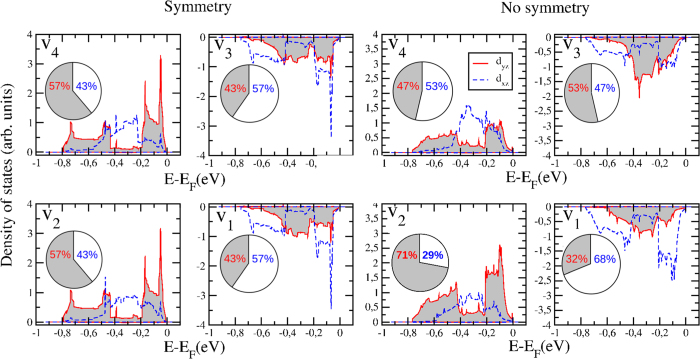
Projected density of states on the d_*yz*_ (grey filled red curve) and d_*xz*_ (unfilled blue dashed curve) orbitals of vanadium by imposing the *Pb*2_1_*m* atomic positions and computing the energy with *Pb*2_1_*m* symmetry (left) for the electronic wavefunction and by removing the symmetry (right) for the electronic wavefunction. *d*_*xy*_ is not displayed for clarity. V_1_ (V_3_) and V_2_ (V_4_) are located within the same (001) plane as defined in Fig. 2. The percentage of total d_*yz*_ (red) and d_*xz*_ (blue) character on each vanadium is shown as a pie chart to illustrate the change from C-o.o to C + G-o.o. once the symmetry constraint for the wavefunction is lifted.

In order to understand the nature of this electronic instability, we plot the projected density of states on vanadiums in Fig. 3. Starting from the projected density of states with *Pb*2_1_*m* symmetry, consecutive atoms along the *z* direction (V_1_ and V_3_, V_2_ and V_4_ on Fig. 2) exhibit identical density of states. Consequently, the orbital ordering appears to be of C-type. When allowing the electronic structure to distort, several changes appear in the orbital occupations. Consecutive atoms along the *z* direction now prefer to occupy either more of the *d*_*xz*_ or the *d*_*yz*_ orbital, which results in a mixed G-type (G-o.o.) plus C-type orbital ordering (C-o.o.). The G-o.o. that appears, despite the absence of the 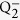 motion, is allowed *via* the Kugel-Khomskii mechanism^44^. This mixed orbital ordering produces an asymmetry between the VO_2_ planes, as indicated by the two magnitudes of magnetic moments in each layer (1.816 ± 0.001 *μ*_*B*_ and 1.819 ± 0.001 *μ*_*B*_). The mixed orbital ordering also appears in the bulk vanadates, such as the G-o.o. + C-o.o. ground state of LaVO_3_ or PrVO_3_ (previously thought to be just G-o.o. from experiments)^25^,^26^. However, here it is not enough to break the inversion symmetry along the *z* axis yielding no out-of-plane polarization. The second necessary ingredient is the symmetry breaking due to the A and A’ ordering along the [001] direction in the superlattices. The combination of both effects (in the AO and VO_2_ planes) is required to break inversion symmetry along the *z* axis and to produce the out-of-plane polarization. The result is an orbital ordering induced ferroelectricity in vanadate superlattices.

Interestingly, the direction of the orbital ordering induced ferroelectric polarization is found to be arbitrary, and both + 0.04 and −0.04 *μC*.*cm*^−2^ are observed. Each state displays a reversal of the magnitude of the magnetic moment of the two VO_2_ planes. Starting from these two possibilities, we performed the geometry relaxation and it ended with the previously identified *Pb* ground states, with both possibilities (up and down) for the out-of-plane polarization. We note that the difference in magnetic moment between both VO_2_ planes is more pronounced (1.820 ± 0.001 *μ*_*B*_ and 1.828 ± 0.001 *μ*_*B*_) after the geometry relaxation. Three new lattice distortions develop to reach the *Pb* phase: *P*_*z*_, 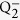 and 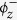.

**Figure 4:**
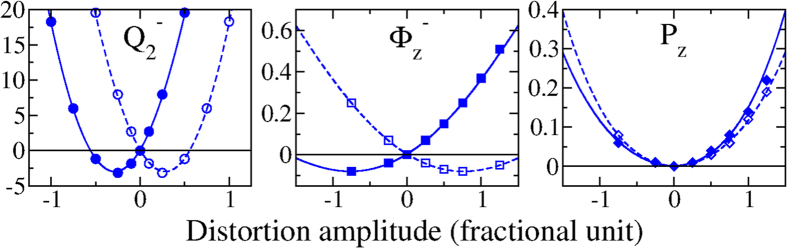
Energy gains (in meV) by condensing various amplitudes of the individual 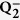, 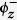 and *P*_*z*_ distortions. Potentials are plotted for a ‘↑’ (open blue symbols) and a ‘↓’ (filled blue symbols) initial electronic polarization.

To understand the nature of their appearance, we plot in Fig. 4 each potential as a function of the distortion amplitude. All potentials present single wells, more or less shifted through an improper coupling with the electronic instability. This confirms that the electronic instability is the primary order parameter driving the phase transition. Moreover, the 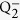 motion presents an energy gain of one to two orders of magnitude larger than those of the 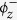 motion, indicating that the 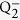 couples more strongly with the electronic instability, which might be expected. Consequently, once the electronic instability condenses, the 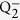 lattice distortion is forced into the system which consequently produces the lattice part of the polarization through the structural hybrid improper coupling. This Jahn-Teller induced ferroelectricity amplifies by one order of magnitude the electronic out-of-plane polarization. The sign of the three lattice distortions is again imposed by the initial sign (up or down) of the electronic polarization. Consequently, the reversal of *P*_*z*_ through an application of an external electric field would require the reversal of both 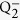, 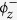 and the magnitude of the magnetic moment of both VO_2_ planes. The saddle point at the midway of this reversal (all three modes equal zero, i.e. the *Pb*2_1_*m* phase) is of the order of 10 meV higher in energy, which represents a reasonable estimate of the ferroelectric switching barrier. Compared to the rotationally driven ferroelectricity *P*_*xy*_, whose energy barrier is of the order of 0.1 to 1 eV^12^,^14^,^15^, this Jahn-Teller induced ferroelectricity is therefore very likely to be switchable. The large difference between the two energy barriers is due to two different energy landscapes involving i) the robust AFD motions inducing *P*_*xy*_ and ii) the relatively soft distortions inducing *P*_*z*_.

Finally we discuss a novel route to create the technologically desired electrical control of magnetization. Starting from a *Pb*2_1_*m* phase with an G-AFM magnetic ordering, the application of an external electric field 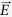 along *z* will induce *P*_*z*_ in the system through the dielectric effect. As a result, the 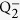 distortion is automatically induced through the 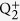 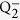 *P*_*z*_ trilinear term. This electric field induced 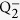 distortion is a general result for any (ABO_3_)_1_/(A’BO_3_)_1_ superlattice consisting of two *Pbnm* perovskites. Since 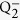 distortions are intimately connected to the G-o.o. and the C-AFM magnetic ordering, for a finite value of 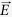, the system may switch from the initial G-AFM phase to the C-AFM phase. In reality, the C-AFM phase should exhibit a net weak magnetization from a non collinear magnetic structure as observed in several bulk vanadates of *P*2_1_/*b* symmetry^28^,^30^,^53^. Therefore, the application of an electric field may not only switch between AFM orderings, but also produce a net magnetic moment in the material. However, for illustrative purposes, even at the collinear level in our calculations, we can look at the relative stability between the two magnetic states under an external electric field in the YLVO superlattice, which presents the desired *Pb*2_1_*m* ground state (8 meV lower than the *Pb* phase).

**Figure 5:**
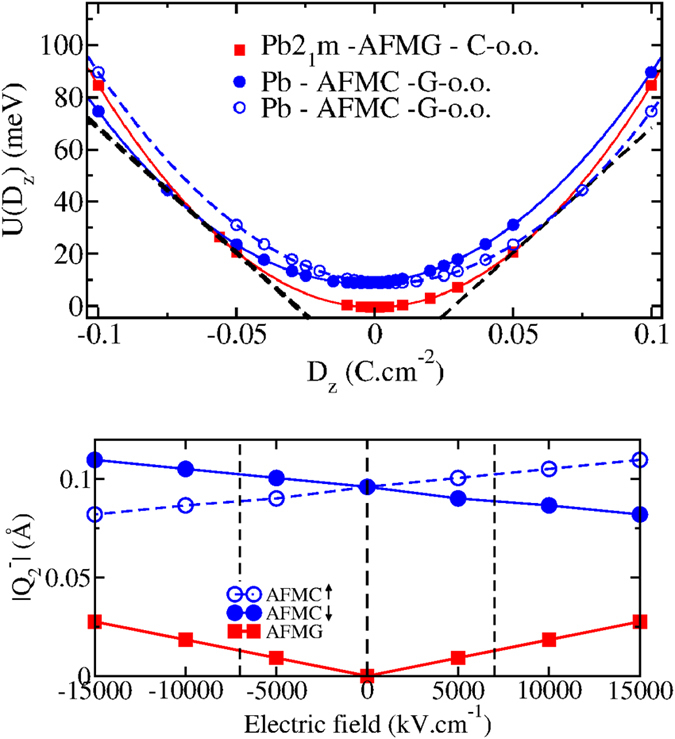
(top) Internal energy U(D) (in meV) as a function of the electric displacement field D along the *z* axis for the different phases of YLVO superlattices. The black dashed line is the common tangent of the two potentials defining the value of the switching electric field. Two polar states are possible for the *Pb* phase starting from an initial up or down *P*_*z*_ polarization. (bottom) Amplitude of 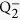 distortion as a function of the electric field along the *z* direction in the *Pb*2_1_*m*-G-AFM phase (red filled squares) and in the *Pb*-C-AFM phases with initial up (blue open circles) and down (blue filled circles) polarisation in zero field.

Figure 5 (top panel) plots the internal energy *U*(*D*) of the *Pb* and *Pb*2_1_*m* phases as a function of the amplitude of the electric displacement field D applied along the *z* axis. In such a graph, the switching E-field at which the the *Pb* phase with the G-o.o./C-AFM ordering becomes more stable than the *Pb*2_1_*m* phase with the C-o.o./G-AFM ordering, is given by the slope of the common tangent between the two curves and is evaluated to be around 6.54 MV.cm^−1^. This corresponds to a voltage of 0.50 V for one bilayer 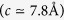. This critical electric field could be further decreased by reducing the energy difference between the two phases at zero field. This can be achieved by changing the A cations or applying biaxial epitaxial strain (see supplementary material).

As illustrated in Fig. 5, what we demonstrate more generally in the present study is an electric field control of the JT distortions, mediated by their coupling with the polar mode. Since this mechanism arises from universal symmetry relations, we can expect this effect to also appear in other perovskite superlattices, such as nickelates, fluorites or manganites to name a few. This effect may find applications outside the field of magnetoelectrics such as for tunable band gaps and metal-insulator transitions, since the JT distortion affects the electronic structure in general.

## Conclusions

In conclusion, we have identified novel lattice mode couplings in the vanadates, helping to clarify the origin of the unusual coexisting Jahn-Teller phase, and indeed the role of Jahn-Teller distortions in perovskites in general. These findings have enabled the prediction of a novel paradigm for the elusive magnetoelectric multiferroics, based on a Jahn-Teller/orbital ordering induced ferroelectricity. Due to the intimate connection between Jahn-Tellers and orbital ordering with magnetism, this unprecedented type of improper ferroelectric facilitates an electric field control of both orbital-ordering and magnetization. The rationale is completely general, and a challenge for applications will be to identify new materials with a magnetic and co-existing Jahn-Teller phase at room temperature. The demonstration of an electric field control of Jahn-Teller distortions may find more general applications for novel functional devices, outside the field of multiferroics. We hope these discoveries will help motivate future studies that will further unlock the potential of vanadate perovskites, and other Jahn-Teller systems, such as fluorites, nickelates and manganites.

## Methods

The basic mechanism we propose here is solely based on symmetry arguments. Symmetry mode analysis of experimental data were performed using amplimodes^46^,^47^. The free energy expansion of Eq. (1 is performed using the invariants software from the isotropy code^58^. The results from these symmetry considerations are not dependent on the technical parameters of the first-principles calculations. The latter are only there to illustrate on a concrete basis and quantify the effect. First principles density functional theory calculations were performed using the VASP package^59^,^60^. We used a 6 × 6 × 4 Monkhorst-Pack k-point mesh to model the *Pbnm* (*P*2_1_/*b*) phase and a plane wave cut off of 500 eV. Optimized Projector Augmented wave (PAW) potentials for PBEsol exchange-correlation functional were used in the calculations. The polarization was computed using the Berry phase approach as implemented in VASP. The study was performed within the LDA + U framework^61^,^62^. The LDA + U framework has already been shown to be sufficient to reproduce the ground state of vanadates^37^,^63^. The U parameter was first fitted on bulk compounds in order to correctly reproduce the ground state of the bulk vanadates. A value of U = 3.5 eV was obtained (see Table 2 and Table 3 in the supporting information). Phonon calculations were performed using the density functional perturbation theory. We used a collinear approach to model the magnetic structures. Structural relaxations were performed until the maximum forces were below 5 *μ*eV.Å^−1^ and the energy difference between conjugate gradient steps was less than 10^−9^ eV. The superlattices were relaxed starting from four different initial guesses: two magnetic orderings (C-AFM and G-AFM) and two space groups (*Pb*2_1_*m* and *Pb*, subgroups of *Pbnm* and *P*2_1_/*b* respectively for the layered structures). Lattice distortion potentials were plotted as a function of the fractional amplitude of each mode separately appearing in the ground state. In order to determine the electric field required to switch from the *Pb*2_1_*m* to the *Pb* phase, the internal energy at fixed D was estimated for each phase as follows. First, the polar atomic distortion pattern *ξ* associated to the linear response of the system to an electric field *E* along *z* was determined from the knowledge of the phonon frequencies and oscillator strengths. Second, the Kohn-Sham energy well *U*_*KS*_(*ξ*) in terms of the amplitude of *ξ* was computed, yielding a model *U*_*KS*_(*P*^0^) restricted to the subspace spanned by *ξ* using *P*^0^ = Z^*^ξ/Ω where Z^*^ is the Born effective charge associated to *ξ*, Ω the unit cell volume. Third, U(D) was deduced as^64^:
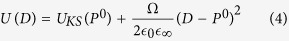
where 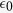 is the permittivity of vacuum and 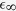, the optical dielectric constant.

## Additional Information

**How to cite this article**: Varignon, J. *et al.* Coupling and electrical control of structural, orbital and magnetic orders in perovskites. *Sci. Rep.* **5**, 15364; doi: 10.1038/srep15364 (2015).

## Supplementary Material

Supplementary Information

## Figures and Tables

**Table 1:** Amplitudes of distortions (in Å) on experimental structures of vanadates at different temperatures.

**Mode (Irreps)**	**YVO3**			**PrVO3**		**LaVO3**	
	***Pbnm***	***P*****21/*****b***	***Pbnm***	***Pbnm***	***P*****21/*****b***	***Pbnm***	***P*****21/*****b***
	**295 K**^51^	**100 K**^36^	**5 K**^51^	**295 K**^26^	**5 K**^26^	**298 K**^52^	**10 K**^23^
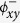 + 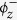 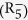	—	1.72	—	—	1.34	—	1.16
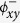 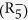	1.71	—	1.73	1.34	—	1.17	—
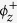 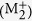	1.22	1.24	1.22	0.94	0.96	0.68	0.75
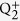 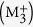	0.05	0.06	0.14	0.02	0.02	0.08	0.01
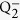 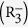	—	0.10	—	—	0.18	—	0.08
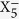 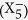	0.86	0.87	0.87	0.53	0.64	0.37	0.39
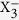 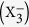	—	0.01	—	—	0.06	—	0.00

In the *P*2_1_/*b* symmetry, both 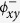 and 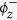 AFD motions belong to the same irreducible representation 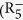 mode) even if the 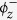 amplitude is likely very small. The reference structure was chosen as a cubic structure whose lattice vector corresponds to the pseudo cubic lattice vector associated to the room temperature *Pbnm* phase.
