# Supplementary material for: Coupling and electrical control of structural, orbital and magnetic orders in perovskites
Source: Sci Rep. 2015 Oct 20;5:15364. doi: 10.1038/srep15364 (PMC4612717; doi:10.1038/srep15364)
Supplement: Supplementary Information [file srep15364-s1.pdf]

Coupling and electrical control of structural, orbital and magnetic orders in perovskites - Supplementary material

Julien Varignon[a,1,†] Nicholas C. Bristowe,[1,2,†] Eric Bousquet,[1] and Philippe Ghosez[1]

[1]*Physique Théorique des Matériaux, Université de Liège (B5), B-4000 Liège, Belgium*[‡]

[2]*Department of Materials, Imperial College London, London SW7 2AZ, UK*

(Dated: February 20, 2015)

[a] corresponding author: julien.varignon@ulg.ac.be

ADDITIONAL LATTICE DISTORTIONS

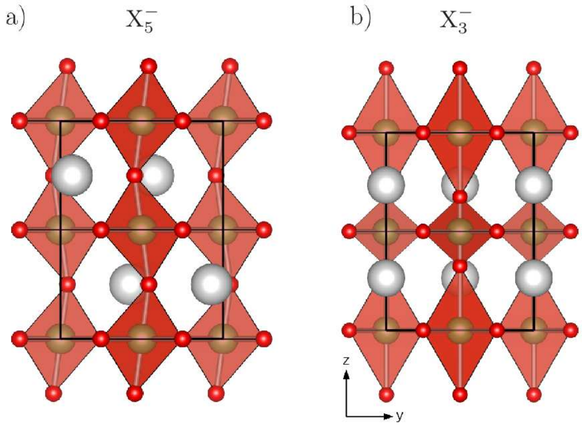

FIG. 1. Additional antipolar motions appearing in the bulk ground states. a) X_5^- anti polar mode appearing in both $Pbnm$ and $P2_1/b$ phases. b) X_3^- antipolar mode appearing exclusively in the $P2_1/b$ phase.

OPTIMIZED BULK VANADATES AT 0K

In order to extract the effective U parameter for our DFT calculations, we fitted its value on bulk vanadates in order to correctly reproduce the ground state of YVO_3, $PrVO_3$ and $LaVO_3$. With an effective parameter of U of 3.5 eV, we correctly reproduce the $Pbnm$ ground state of YVO_3 and the $P2_1/b$ ground state of both $PrVO_3$ and $LaVO_3$. Optimized lattice parameters and symmetry mode analysis are given in Tables I and II respectively. Within our DFT calculations, YVO_3 exhibits a gap of 1.95 eV in close comparison with experimental value (around 1.6 eV) [1], while $LaVO_3$ and $PrVO_3$ develop band gaps of 1.70 eV and 1.93 eV respectively, slightly overestimated [2].

OPTIMIZED $(AVO_3)_1/(A'VO_3)_1$ LAYERED STRUCTURES

Symmetry mode analysis

Symmetry mode analysis of our optimized vanadate layered structures are provided in Table III. The superlattice is insulating and develops a band gap of 1.86 eV.

Origin of P_{xy}

As discussed in the main paper, bulk vanadates develop a $Pbnm$ symmetry at room temperature and hence the layered structures should present the equivalent $Pb2_1m$ symmetry. In the $Pb2_1m$ phase, four main distortions are present: ϕ_{xy}^-, ϕ_z^+, Q_2^+ and P_{xy}. In order to understand the driving force leading to this metastable $Pb2_1m$ symmetry, we added some amplitude of distortions leading to this symmetry in a hypothetical $P4/mmm$ phase. Potentials are plotted in Figure2. As expected, the two AFD motions present double wells and are strongly unstable (energy gains around 1 eV) while P_{xy} and Q_2^+ are stable. Consequently, ϕ_{xy}^- and ϕ_z^+ are the primary order parameters leading to the $Pb2_1m$ symmetry, and P_{xy} and Q_2^+ appear through improper couplings. These potentials confirm the hybrid improper character of the in-plane P_{xy} polarization [6–8].

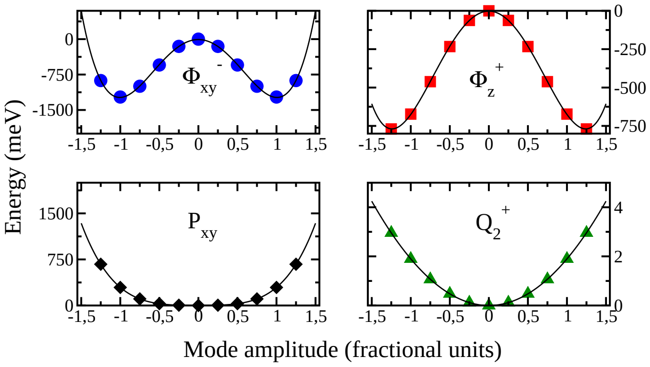

FIG. 2. Energy gains (in meV) by condensing several amplitudes of distortions leading to the $Pb2_1m$ (AFMG) within the P_4/mmm structure.

STRAIN EFFECT ON $(AVO_3)_1/(A'VO_3)_1$ LAYERED STRUCTURES

For potential applications, the proposed superlattices can be grown on given substrates. The pseudocubic parameters (defined as the mean value of the low symmetry lattice parameters) are 3.88 Å, 3.88 Å and 3.92 Å for YLVO and PLVO superlattices respectively. We chose cubic $SrTiO_3$ (3.905 Å) and $KTaO_3$ (3.9885 Å) as two potential substrates and performed a geometry relaxation of the two superlattices constraining both in-plane pseudocubic lattice parameters to those of the substrates [9]. Results are displayed in Table IV. The effect of the substrates is to tune the relative energy difference between the two magnetic orderings, and hence between the two orbital-ordered phases. A $SrTiO_3$ substrate seems to favor an AFMG magnetic ordering, and consequently a pure Q_2^+ phase. On the contrary, going to a $KTaO_3$ substrate applies a moderate tensile strain and favors an AFMC magnetic ordering for the two superlattices. These strain effects then open the way to an indirect control of the magnetism and the orbital ordering playing with the strain imposed by the substrates. Indeed, growing $(AVO_3)_1/(A'VO_3)_1$ layered structures on a piezoelectric substrates, an indirect coupling with the electric field may appear:

$$\text{coupling} \propto \frac{electric}{strain} \times \frac{strain}{magnetism}$$

Applying an external field on the substrate induces a strain on the vanadate layered structure, and consequently this latter can switch both magnetic and orbital orderings.

---

† These two authors contributed equally
‡ julien.varignon@ulg.ac.be
[1] Tsvetkov, A. *et al.* Structural, electronic, and magneto-optical properties of YVO_3. *Phys. Rev. B* **69**, 075110 (2004).
[2] Arima, T., Tokura, Y., Torrance, J. Variation of optical gaps in perovskite-type 3d transition-metal oxides. *Phys. Rev. B* **48**, 17006 (1993).
[3] Reehuis, M. *et al.* Neutron diffraction study of YVO_3, $NdVO_3$, and $TbVO_3$. *Phys. Rev. B* **73**, 094440 (2006).
[4] Sage, M., Blake, G., Marquina, C., Palstra, T. Competing orbital ordering in RVO_3 compounds: High-resolution X-ray diffraction and thermal expansion. *Phys. Rev. B* **76**, 195102 (2007).
[5] Bordet, P. *et al.* Structural aspects of the crystallographic-magnetic transition in $LaVO_3$ around 140 K. *J. Solid State Chem.* **106**, 253-270 (1993).
[6] Bousquet, E. *et al.* Improper ferroelectricity in perovskite oxide artificial superlattices. *Nature* **452**, 732-736 (2008).
[7] Rondinelli, J. M., Fennie, C. J. Octahedral rotation-induced ferroelectricity in cation ordered perovskites. *Adv. Materials* **24**, 1961-1968 (2012).
[8] Fukushima, T., Stroppa, A., Picozzi, S., Perez-Mato, J. M. Large ferroelectric polarization in the new double perovskite $NaLaMnWO_6$ induced by non-polar instabilities. *Phys. Chem. Chem. Phys.* **13**, 12186-12190 (2011).
[9] Schlom, D. G. *et al.* Strain tuning of ferroelectric thin films. *Annu. Rev. Mater. Res.* **37**, 589-626 (2007).

		YVO$_3$		PrVO$_3$	LaVO$_3$
		Pbnm	$P2_1/b$	$P2_1/b$	$P2_1/b$
		AFMG	AFMC	AFMC	AFMC
a(Å)	th.	5.28	5.27	5.48	5.55
	exp.	5.29(5K) [3]	5.28(85K) [3]	5.48(5K) [4]	5.56(10K) [5]
b (Å)	th.	5.61	5.65	5.68	5.64
	exp.	5.59(5K) [3]	5.62(85K) [3]	5.61(5K) [4]	5.59(10K) [5]
c (Å)	th.	7.58	7.55	7.72	7.76
	exp.	7.56(5K) [3]	7.54(85K) [3]	7.69(5K) [4]	7.75(10K) [5]
α (°)	th.	-	90.03	90.14	90.15
	exp.	-	90.02(85K) [3]	90.15(5K) [4]	90.13(10K) [5]

TABLE I. Optimized lattice parameters for the bulk vanadates at 0 K. Experimental values are given for comparison.

Mode (Irreps)	YVO$_3$ *Pbnm*	YVO$_3$ $P2_1/b$	PrVO$_3$ $P2_1/b$	LaVO$_3$ $P2_1/b$
ϕ_{xy}^- (+ ϕ_z^-) (R$_5^-$)	-	1.81	1.42	1.32
ϕ_{xy}^- (R$_5^-$)	1.83	-	-	-
ϕ_z^+ (M$_2^+$)	1.24	1.25	0.97	0.89
Q$_2^+$ (M$_3^+$)	0.15	0.06	0.02	0.01
Q$_2^-$ (R$_3^-$)	-	0.10	0.10	0.09
X$_5^-$ (X$_5^-$)	0.90	0.90	0.65	0.52
X$_3^-$ (X$_3^-$)	-	0.05	0.01	0.00

TABLE II. Amplitudes of distortions (in Å) on our optimized bulk vanadates at 0 K with respect to a hypothetical pseudocubic phase. In the $P2_1/b$ symmetry, both ϕ_{xy}^- and ϕ_z^- belong to the same irreps, even if ϕ_z^- amplitude of distortion should remain extremely small.

Mode (Irreps)	YLVO (0K) $Pb2_1m$-AFMG	PLVO (0K) Pb-AFMC
ϕ_{xy}^- (M$_5^-$)	1.58	1.36
ϕ_z^+ (M$_2^+$)	1.13	0.94
ϕ_z^- (M$_4^-$)	-	0.01
M$_{JT}$ (M$_3^+$)	0.13	0.01
R$_{JT}$ (M$_1^-$)	-	0.09
P$_{xy}$ (Γ_5^-)	0.76 (7.89 $\mu C.cm^{-2}$)	0.59 (2.94 $\mu C.cm^{-2}$)
P$_z$ (Γ_3^-)	- -	0.00(4) (0.34 $\mu C.cm^{-2}$)

TABLE III. Amplitudes of distortions (in Å) on our optimized bulk vanadates at 0 K with respect to a hypothetical cube on cube phase ($P4/mmm$). In the $P2_1/b$ symmetry, both ϕ_{xy}^- and ϕ_z^- belong to the same irreps, even if ϕ_z^- amplitude of distortion should remain extremely small.

		$SrTiO_3$		Fully relaxed		$KTaO_3$	
		AFMG	AFMC	AFMG	AFMC	AFMG	AFMC
YLVO	ΔE	0	+24	0	+8	0	-10
	c/a	0.98	0.98	1.01	1.01	0.95	0.95
PLVO	ΔE	0	+8	0	-4	0	-13
	c/a	1.00	1.00	1.00	1.00	0.96	0.96

TABLE IV. Amplitudes of distortions (in Å) on our optimized bulk vanadates at 0 K with respect to a hypothetical pseudocubic phase. In the $P2_1/b$ symmetry, both ϕ_{xy}^- and ϕ_z^- belong to the same irreps, even if ϕ_z^- amplitude of distortion should remain extremely small.
